# Engineering *Sphingobium* sp. to Accumulate Various Carotenoids Using Agro-Industrial Byproducts

**DOI:** 10.3389/fbioe.2021.784559

**Published:** 2021-11-04

**Authors:** Mengmeng Liu, Yang Yang, Li Li, Yan Ma, Junchao Huang, Jingrun Ye

**Affiliations:** ^1^ School of Marine Science and Engineering, Qingdao Agricultural University, Qingdao, China; ^2^ Key Laboratory of Microbial Technology, Shandong University, Qingdao, China; ^3^ Qingdao Eighth People’s Hospital, Qingdao, China; ^4^ Department of Laboratory Medicine, Qingdao Central Hospital, Qingdao, China; ^5^ Institute for Advanced Study, Shenzhen University, Shenzhen, China

**Keywords:** *Sphingobium*, crop wastes, carotenoids, biosynthetic pathway, astaxanthin

## Abstract

Carotenoids represent the most abundant lipid-soluble phytochemicals that have been shown to exhibit benefits for nutrition and health. The production of natural carotenoids is not yet cost effective to compete with chemically synthetic ones. Therefore, the demand for natural carotenoids and improved efficiency of carotenoid biosynthesis has driven the investigation of metabolic engineering of native carotenoid producers. In this study, a new *Sphingobium* sp. was isolated, and it was found that it could use a variety of agro-industrial byproducts like soybean meal, okara, and corn steep liquor to accumulate large amounts of nostoxanthin. Then we tailored it into three mutated strains that instead specifically accumulated ∼5 mg/g of CDW of phytoene, lycopene, and zeaxanthin due to the loss-of-function of the specific enzyme. A high-efficiency targeted engineering carotenoid synthesis platform was constructed in *Escherichia coli* for identifying the functional roles of candidate genes of carotenoid biosynthetic pathway in *Sphingobium* sp. To further prolong the metabolic pathway, we engineered the *Sphingobium* sp. to produce high-titer astaxanthin (10 mg/g of DCW) through balance in the key enzymes β-carotene ketolase (BKT) and β-carotene hydroxylase (CHY). Our study provided more biosynthesis components for bioengineering of carotenoids and highlights the potential of the industrially important bacterium for production of various natural carotenoids.

## Introduction

Carotenoids play essential roles in light harvesting and photoprotection in photosynthetic organisms (Niyogi et al., 1997; Niyogi et al., 2001). With some exception, animals and humans cannot synthesize carotenoids *de novo* but take them from the diets, serving as precursors to vitamin A and macula pigments (Grumet et al., 2016). Carotenoids have been applied in food, feed, nutraceuticals, cosmetics, and pharmaceuticals (Cezare-Gomes et al., 2019; Ye et al., 2019). The pathway of carotenoid biosynthesis has been extensively studied in various organisms (Shumskaya and Wurtzel, 2013). The condensation of two geranylgeranyl diphosphate (GGPP) molecules was catalyzed by phytoene synthase (CrtB) to phytoene (C40), which is subsequently desaturated to produce lycopene by phytoene desaturase (CrtI). Next, lycopene was conferred diverse functional groups *via* various carotenoid-modifying enzymes, including lycopene cyclase (CrtY), carotene ketolase (CrtW), and carotene hydroxylase (CrtZ). The cyclic carotenoids derived from lycopene have diverse biological properties and functions (Kim et al., 2014).

Currently, commercial natural carotenoids (e.g., β-carotene, astaxanthin, and zeaxanthin) are extracted from a small number of specific plant tissues and a few microbes (Liu et al., 2019). Microbial production is the major source of natural-origin carotenoids, such as β-carotene and astaxanthin, because of higher growth rates and contents (Zhang et al., 2018). Most studies have mainly focused on the metabolic engineering of the noncarotenogenic *Escherichia coli* and yeast for carotenoid production (Park et al., 2018; Ma et al., 2019). However, the expression of a large number of heterologous genes commonly leads to the instability of the engineered strains because of the consumption of cellular metabolites and the influence on the metabolic flux distribution (Li and Huang, 2018). Engineering with few heterologous genes in the natural carotenogenic microorganism might overcome the above problems. In previous studies, a few attempts have been made to modify the biosynthetic pathway of carotenoids in microorganisms, which has the synthetic pathway of carotenoids, including *Synechocystis* sp., *Xanthophyllomyces dendrorhous*, and *Mucor circinelloides*. However, the yields of the target carotenoids are unsatisfactory (Lagarde et al., 2000; Papp et al., 2006; Breitenbach et al., 2019).

Now, the production capacity of carotenoids in wild-type microbes, by improving fermentation efficiency, is still unsatisfactory. The main challenge in carotenoid production using microbes is to develop new resources that could use cost-effective culture medium to produce high carotenoid content and microbial biomass (Rodrigues et al., 2019). A feasible strategy for meeting the challenge is to construct high-yield carotenoid microorganism by metabolic engineering that could use agro-industrial byproducts for reducing production cost and maintaining sustainable development. Glycerol, corn steep, and okara have attracted interest as raw materials for microbial to production of value-added chemicals and compounds (Liu et al., 2012a; Lee et al., 2019; Zhao et al., 2019; Kang et al., 2020). Crude glycerol is a byproduct generated from biodiesel production; okara and corn steep liquor are generated as byproducts during the manufacture of soymilk and corn starch. Although glycerol, okara, and corn steep liquor are considered as byproducts, they are nutrient rich. However, they are highly underused energy sources, as a large proportion is dumped into incinerators and landfills (Lee et al., 2019; Kang et al., 2020; Zhao et al., 2020).

*Sphingobium* is a representative genus of sphingomonads that are able to synthesize a number of carotenoids, including β-carotene and its derivatives zeaxanthin, astaxanthin, and nostoxanthin (Jenkins et al., 1979; Silva et al., 2004; Ma et al., 2016). Several studies have reported that the sphingomonad families could efficiently degrade environmental pollutants and biowaste (Liu et al., 2012b; Chen et al., 2014; Mishra et al., 2017). Furthermore, this family has been identified as safe (GRAS), and some members have been approved by the USA and the EU to synthesize gellan gum, an extracellular polysaccharide, which is a suspending agent, gelling, and stabilizing for a wide range of foods (Morris, 1990; Sutherland, 1998). Thus, the nonpathogenic and carotenogenic *Sphingobium* bacteria have potential as novel producers of various food-grade carotenoids.

The aim of this study is to target engineer a newly isolated nostoxanthin-accumulated strain *Sphingobium* sp. KIB that could use a variety of crop wastes to four novel strains, which accumulated large amounts of phytoene, lycopene, zeaxanthin, and astaxanthin, respectively. These results indicated that the *Sphingobium* strains have the potential of industrial production of carotenoids.

## Materials and Methods

### Bacterial Strains and Culture Conditions

*Sphingobium* sp. KIB was stored in the China General Microbiological Culture Collection Center (CGMCC No.12394), which was isolated from Kunming Institute of Botany (Liu et al., 2019). *Sphingobium* sp. KIB and its mutants and engineered strains were cultured in basal salt medium (BSM) at 28°C with shaking (220 rpm). BSM medium contains 4 g of (NH_4_)_2_HPO_4_, 10 g of glucose, 0.585 g of MgSO_4_, 13.3 g of KH_2_PO_4_, 1.86 g of citric acid monohydrate, and 10 ml of trace element solution per liter. The trace element solution contains 15 mg of CuCl_2_·2H_2_O, 25 mg of CoCl_2_·6H_2_O, 25 mg of NaMoO_4_·2H_2_O, 30 mg of H_3_BO_3_, 84 mg of disodium EDTA·H_2_O, 80 mg of Zn (CH_3_COO)_2_·2H_2_O, 123 mg/L of MnCl_2_·2H_2_O, and 600 mg of ferric citrate. The pH value was adjusted with KOH. For testing of the carbon sources or nitrogen source utilization, the glucose or (NH_4_)_2_HPO_4_ was replaced by 10 g/L of other carbon sources or 4 g/L of other nitrogen sources. *E. coli* JM109 was cultured at 37°C in LB medium and was used to clone expressing constructs for various carotenoids. Ampicillin (100 µg/ml) and/or chloramphenicol (34 µg/ml) was used when required.

### Mutagenesis and Mutant Selection

*Sphingobium* sp. KIB was cultivated to a cell density of 0.7–0.8 measured by OD600. The bacterial cells (20 ml) were pelleted by centrifugation at 4,000 × *g* for 3 min and washed twice with 20 ml of PBS buffer. The cells were resuspended in 20 ml of LB medium and then treated with N-methyl-N-nitro-nitrosoguanidine (MNNG) in a final concentration of 3.5 mM for 1 h in the dark. Following the mutagenesis treatment, cells were washed with the growth medium four times to dilute mutagen and then incubated 6 h on a shaker (28°C, 150 rpm). Subsequently, cells were plated on LB plates, and colonies were developed at 28°C for 3 days. Colonies were screened based on their pigmentation (Li et al., 2017). Carotenoids appear to be different in colors, e.g., the colorless phytoene, red lycopene, and yellow zeaxanthin. Thus, mutants could be isolated by visual color screening. Colonies with different colors from wild-type strain were selected for further analysis of pigment composition.

### Molecular Characterization of Mutants

DNA isolation were carried out according to Ma et al. (2016). Based on the genome sequence (accession No. SRR8864026) of *Sphingobium* sp. KIB, primers (Supplementary Table S1) were designed to amplify the full length of CrtB, CrtI, CrtY, and CrtG genes. The PCR programs were list in Supplementary Table S1. PCR products were gel purified and sequenced. The Bio Edit software was used for sequence alignment.

### Growth and Biomass Measurement

Exponential-phase cells were inoculated into 50 ml of culture medium in 250-ml Erlenmeyer flasks. Cultures were incubated at 28°C and 220 rpm in an orbital shaker. Samples were collected at an interval of 6 h for the determination of dry cell weight (DCW). For this, 2 ml of culture was washed twice with distilled water and then filtered through preweighted 0.22-µm membrane filters. Filtered cells were dried to a constant weight in an oven at 65°C.

### Engineering Carotenoid Synthesis Platform in *Escherichia coli*

Recombinant DNA techniques were performed using standard methods. The plasmids and primers used in this study are listed in Supplementary Table S1. Relevant structures of the plasmids are listed in Supplementary Figure S1. The full length of CrtB, CrtI, CrtY, and CrtG genes were cloned, respectively, using *Sphingobium* sp. KIB genome DNA as a template and cloned into the plasmid pACYC184 to create plasmid pACCARcrtEB, pACCARcrtEBI, pACCARcrtEBIYZ, or pACCARcrtEBIYZG. *E. coli* JM109 carrying plasmid pACCARcrtEB, pACCARcrtEBI, pACCARcrtEBIYZ, or pACCARcrtEBIYZG displayed a white, red, yellow, or deep yellow phenotype due to the synthesis of phytoene, lycopene, zeaxanthin, or nostoxanthin.

### Engineering *Sphingobium* sp. to Produce Astaxanthin

The plasmids pSPRGelb-DXS/IDI/CHY/CrtW, pSPRGelb-DXS/IDI/CHY/BKT, pSPRGelb-DXS/IDI/CrtZ/CrtW, and pSPRGelb-DXS/IDI/CrtZ/BKT were constructed according to Liu et al. The gene *CHY* was from *Haematococcus pluvialis*, and the gene *CrtZ* was from *Brevundimonas* sp. SD212. These two genes were cloned and then inserted into plasmid pSPRGelb-DXS/IDI at the SalI site, respectively, to construct the plasmids pSPRGelb-DXS/IDI/CHY and pSPRGelb-DXS/IDI/CrtZ. The gene *BKT* was from *Chlamydomonas reinhardtii*, and the gene *CrtW* was from *Brevundimonas* sp. SD212, and these two genes were cloned and then inserted into plasmids pSPRGelb-DXS/IDI/CHY and pSPRGelb-DXS/IDI/CrtZ at the SalI site, respectively, to construct the plasmids pSPRGelb-DXS/IDI/CHY/CrtW, pSPRGelb-DXS/IDI/CHY/BKT, pSPRGelb-DXS/IDI/CrtZ/CrtW, and pSPRGelb-DXS/IDI/CrtZ/BKT. Then these four plasmids were transformed using electrotransformation into *Sphingobium* sp. KIB-zea (Liu et al., 2019).

### Extraction and Measurement of Carotenoids

Carotenoid analysis was performed according to Liu et al. (2019). Acetone was used to extract carotenoids. The acetone dissolved the carotenoids, which were blown to dryness with nitrogen gas and dissolved in 100 µl of acetone. Carotenoids were analyzed and quantitated using an Agilent Ultra-Performance Liquid Chromatography (UPLC) 1290 Infinity system equipped with an Agilent Eclipse Plus C18 RRHD 1.8-µm column (2.1 × 50 mm). The mobile phase consisted of solvent A (15% methanol, 60% acetonitrile, 5% isopropanol, and 20% water) and solvent B (15% methanol, 5% isopropanol, and 80% acetonitrile). The extracted carotenoids were eluted at a flow rate of 0.5 ml/min with the following process: 100% A for min; a liner gradient from 0 to 100% within 1 min; 100% B for 8 min. Compounds were detected at 454 and 280 nm, and the absorption spectra, retention times, and peak area of each carotenoid were compared with standards purchased from Sigma (China).

### Phylogenetic Analysis

The standard chloroform/isopropanol method was used to extract genomic DNA from *Sphingobium* sp. KIB. Two primers, 27F (5′-AGA​GTT​TGA​TCC​TGG​CTC​AG-3′) and 1429R (5′-GGT​TAC​CTT​GTT​ACG​ACT​T-3′) were used for amplification of the 16S rRNA gene. The amplification product was purified and sequenced by Shenggong Bioscience Company (Shanghai, China). The 16S rRNA gene sequence of *Sphingobium* sp. KIB was submitted to GenBank (accession number MK088252). The phylogenetic tree was constructed using the neighbor-joining method *via* the MEGA 7.0 software.

## Results and Discussions

### Isolation of a Nostoxanthin-Accumulating *Sphingobium* Strain

**FIGURE 1:**
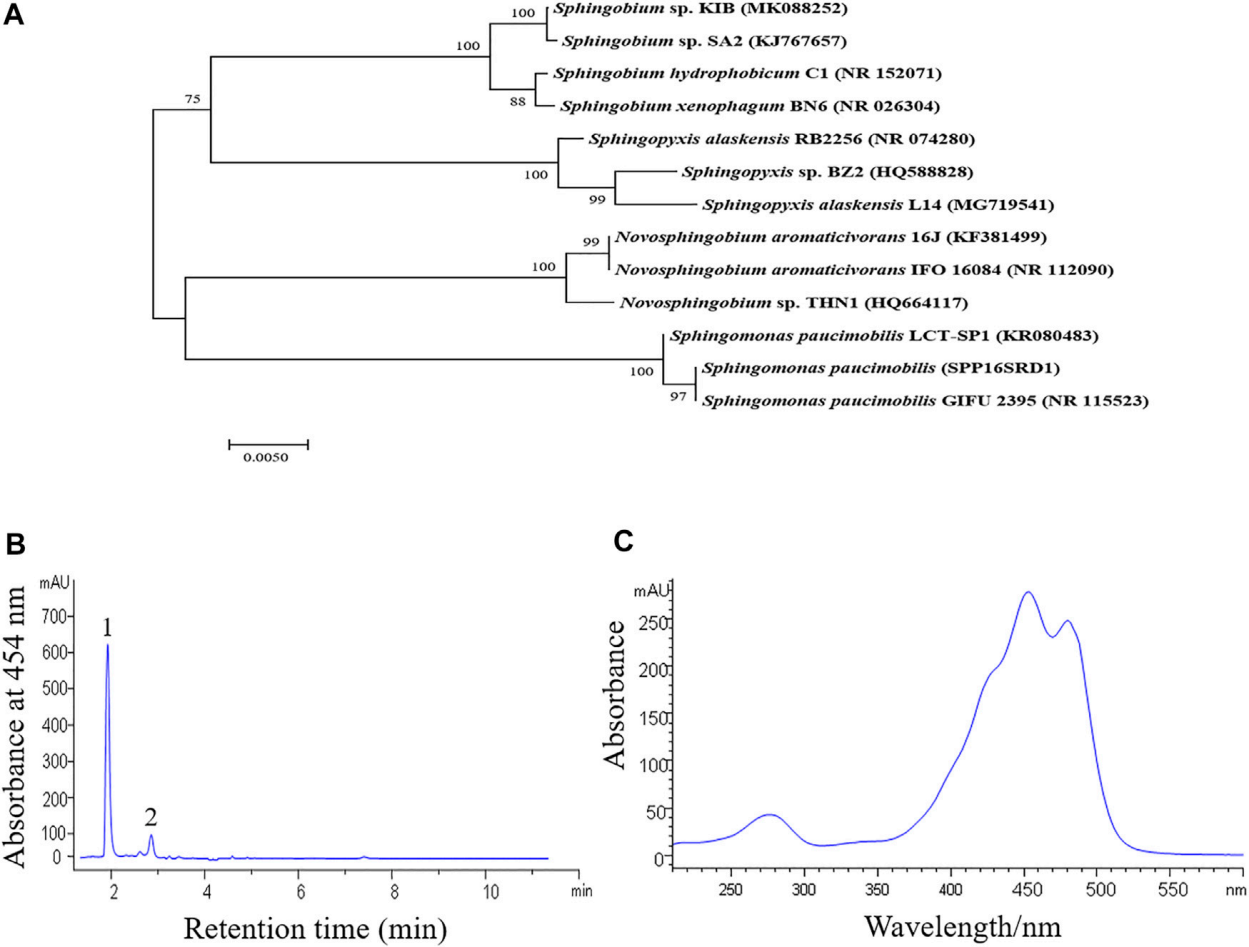
Phylogenetic and carotenoid analysis of *Sphingobium* sp. KIB. **(A)** A phylogenetic tree based on the 16S rRNA gene sequences of *Sphingobium* sp. KIB and its related taxa. The tree was constructed by the neighbor-joining method. Numbers at nodes indicated bootstrap percentages (based on 1,000 resampled datasets). **(B)** Absorption profile of carotenoids extracted from the strain KIB. 1. Nostoxanthin, 2. Caloxanthin. **(C)** Absorption spectrum of peak 1.

A yellow pigmented bacterial strain was isolated from tissue culture plates at Kunming Institute of Botany (KIB), which was revealed to be a rod-shaped and Gram-negative bacterium. Phylogenetic analysis based on the 16S rRNA gene sequences showed that the strain had the highest similarity to *Sphingobium* sp. SA2 (KJ767657.1) (Figure 1A). We, therefore, named the strain as *Sphingobium* sp. KIB. *Sphingobium* sp. KIB was found to produce nostoxanthin, a zeaxanthin derivative, up to 93% of total carotenoids (Figures 1B,C), which was much higher than the levels reported in its relatives (Jenkins et al., 1979; Zhu et al., 2012). We hypothesized that *Sphingobium* sp. KIB was a promising carotenoid-producing strain.

### Utilization of Agro-Industrial Byproducts to Accumulate Carotenoids

**FIGURE 2:**
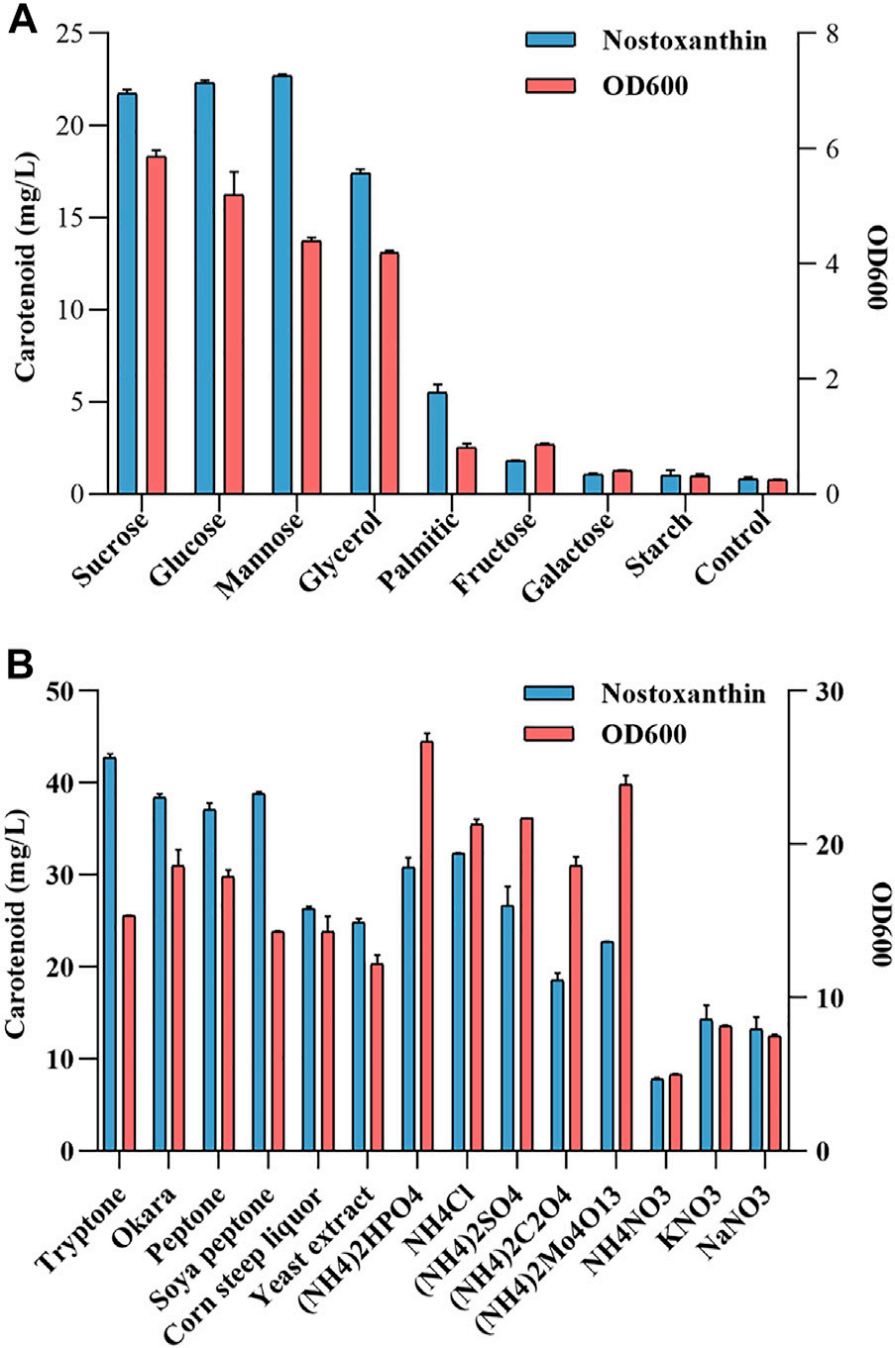
Testing the carbon source **(A)** or nitrogen source **(B)** utilization of the *Sphingobium* sp. KIB.

Various carbon sources or nitrogen sources were tested as a potential nutrient for the fermentation of the *Sphingobium* sp. KIB. It was observed that most carbon sources and nitrogen sources were able to effectively support the growth of *Sphingobium* sp. KIB (Figure 2). Of the eight carbon sources tested, sucrose, glucose, mannose, and glycerol demonstrated the best carbon sources for carotenoid production and cell growth (Figure 2A). Of the 14 nitrogen sources investigated, tryptone, soya peptone, and okara were found to be the best one for carotenoid production, and (NH_4_)_2_HPO_4_ was found to be the best one for cell growth (Figure 2B). These results demonstrated that the agro-industrial byproducts, such as glycerol, okara, and corn steep liquor, could be effectively used by *Sphingobium* sp. KIB to benefit cell growth and stimulate carotenoid accumulation. So, at the following studies, 10 g/L of glycerol was used as carbon source and 4 g/L okara was used as nitrogen source.

These byproducts were highly underused energy sources, as a large proportion is dumped into incinerators and landfills (Lee et al., 2019; Kang et al., 2020). It would be desirable to find that *Sphingobium* sp. KIB could use these agro-industrial byproducts to produce high-value carotenoids. Hence, *Sphingobium* sp. KIB could serve as a starting strain for the efficient production of zeaxanthin (Liu et al., 2019) and possibly other carotenoids using agro-industrial byproducts *via* knocking out or introducing the key enzymes involved in the carotenoid biosynthetic pathway.

### Isolation of *Sphingobium* sp. KIB Mutants Accumulating Other Carotenoids

**FIGURE 3:**
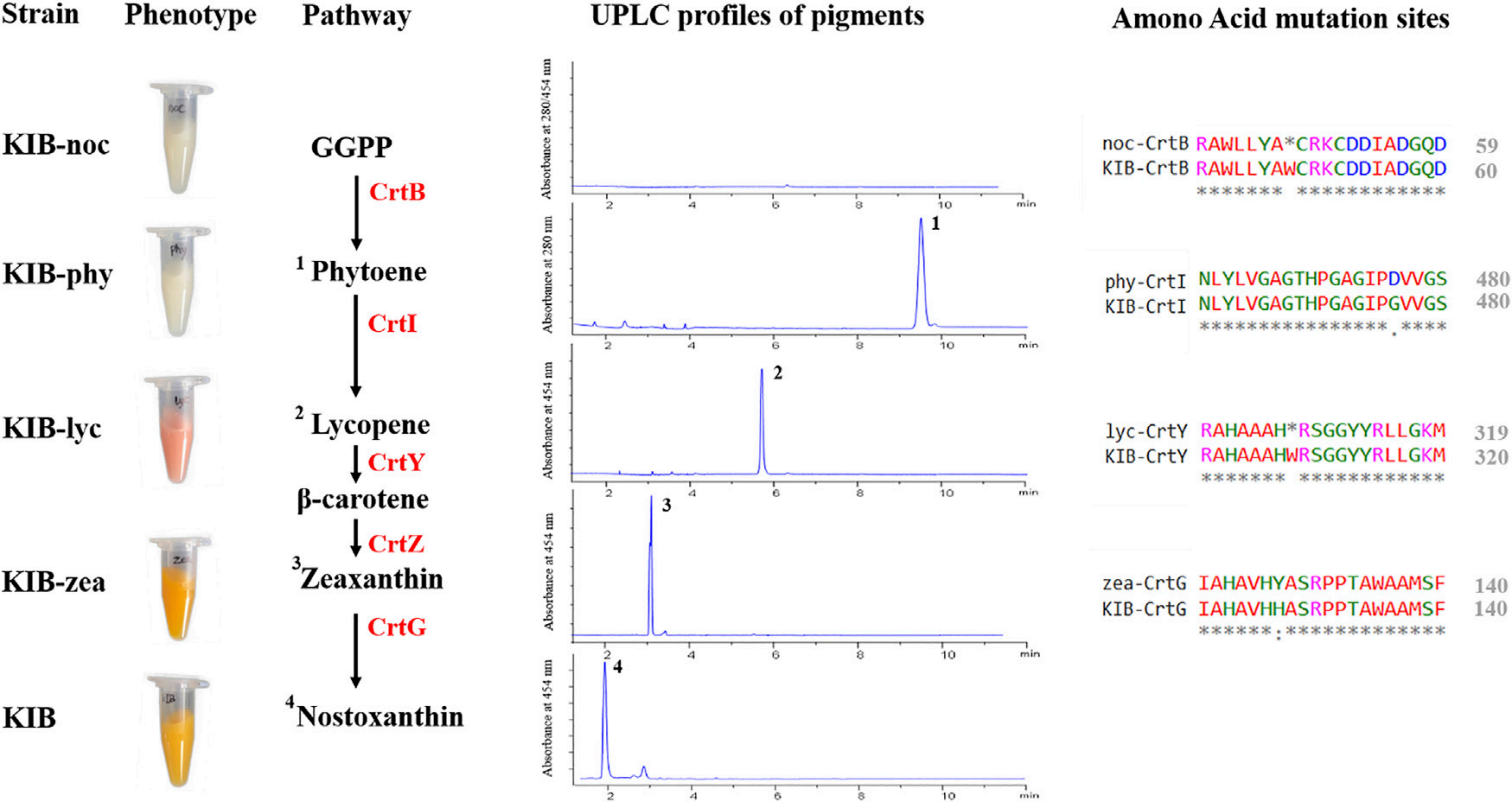
Relevant information of the four mutants. The phenotypes, carotenoid biosynthetic pathway, carotenoid chromatograms, and amino acid substitutions of the mutated targets are shown. 1. Phytoene. 2. Lycopene. 3. Zeaxanthin. 4. Nostoxanthin. GGPP, geranylgeranyl-pyrophosphate; CrtB, phytoene synthase; CrtI, phytoene desaturase; CrtY, lycopene beta-cyclase; CrtZ, beta-carotene hydroxylase; CrtG, 2,2′-beta-ionone hydroxylase.

*Sphingobium* sp. KIB was treated with a chemical mutagen (MNNG) followed by a color-based screening process. About 100 colonies exhibiting different colors from wild-type strain were selected and subjected to pigment analysis. Four stable mutants, accumulating no carotenoid, phytoene, lycopene, or zeaxanthin, were successfully achieved based on their pigment profiles (Figure 3). *Sphingobium* sp. KIB-noc demonstrated a white color and accumulated no carotenoid due to a nonsense mutation in the *CrtB* gene that encodes phytoene synthase involved in catalyzing the first step of carotenoid formation (Figure 3). *Sphingobium* sp. KIB-phy also demonstrated a white color but predominantly accumulated the colorless carotenoid phytoene (up to 90% of total carotenoids) owing to one amino acid substitution (G476D) in the CrtI (Figure 3). *Sphingobium* sp. KIB-lyc exhibited a red color and mainly accumulated lycopene due to the nonsense mutation of CrtY, which is involved in converting lycopene to β-carotene (Figure 3). *Sphingobium* sp. KIB-zea was yellow and accumulated zeaxanthin up to 90% of total carotenoids, which consisted of an amino acid substitution (H127Y) in CrtG; the enzyme catalyzes zeaxanthin to nostoxanthin (Figure 3).

**FIGURE 4:**
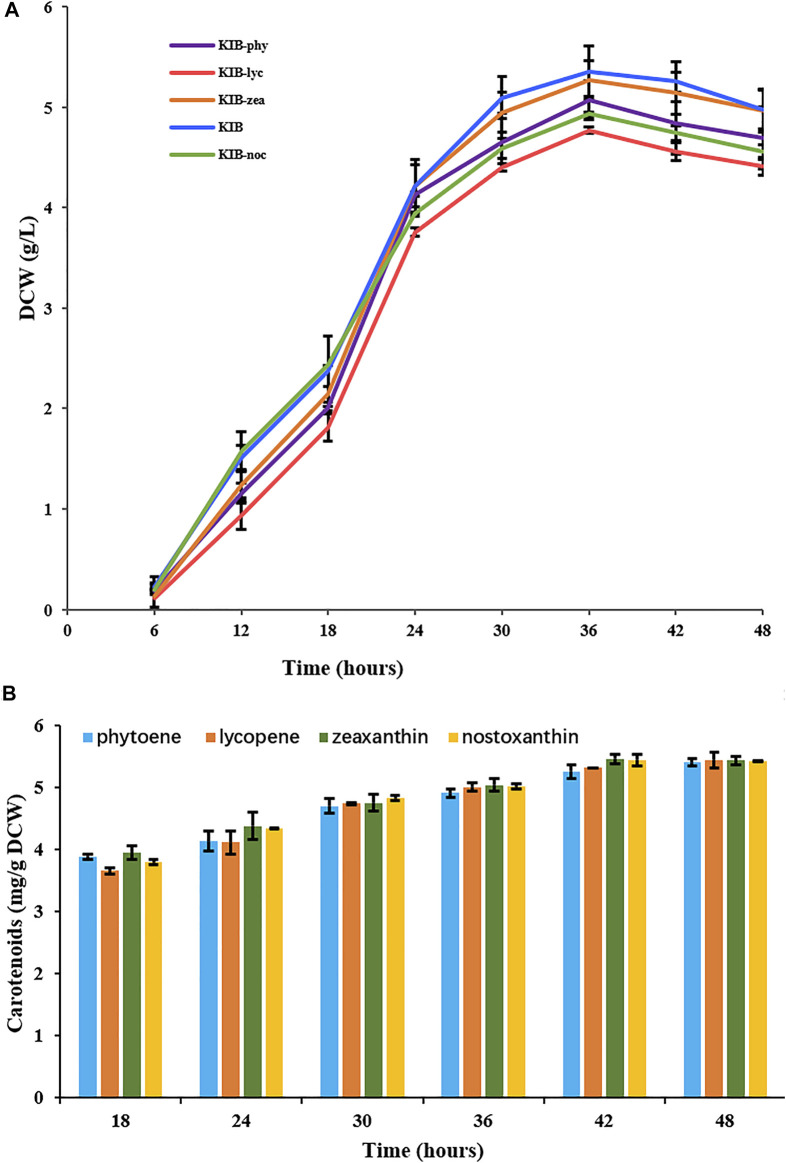
Growth **(A)** and carotenoid contents **(B)** of *Sphingobium* sp. KIB and its mutants. KIB produced nostoxanthin, KIB-phy produced phytoene, KIB-lyc produced lycopene and KIB-zea produced zeaxanthin.

The four mutants showed similar growing status to their parent wild-type strain (Figure 4A). All strains reached their highest biomass at 36 h. Wild type and the mutants KIB-phy, KIB-lyc, and KIB-zea constitutively accumulated nostoxanthin, phytoene, lycopene, and zeaxanthin, respectively. The contents of the carotenoids in the strains increased over time, reaching the highest value of about 5.4 mg g^−1^ of dry cell weight at 48 h (Figure 4B). This result indicated that *Sphingobium* sp. KIB and its mutants maintained a homeostasis of carotenoids, irrespective of the kinds of carotenoids.

**FIGURE 5:**
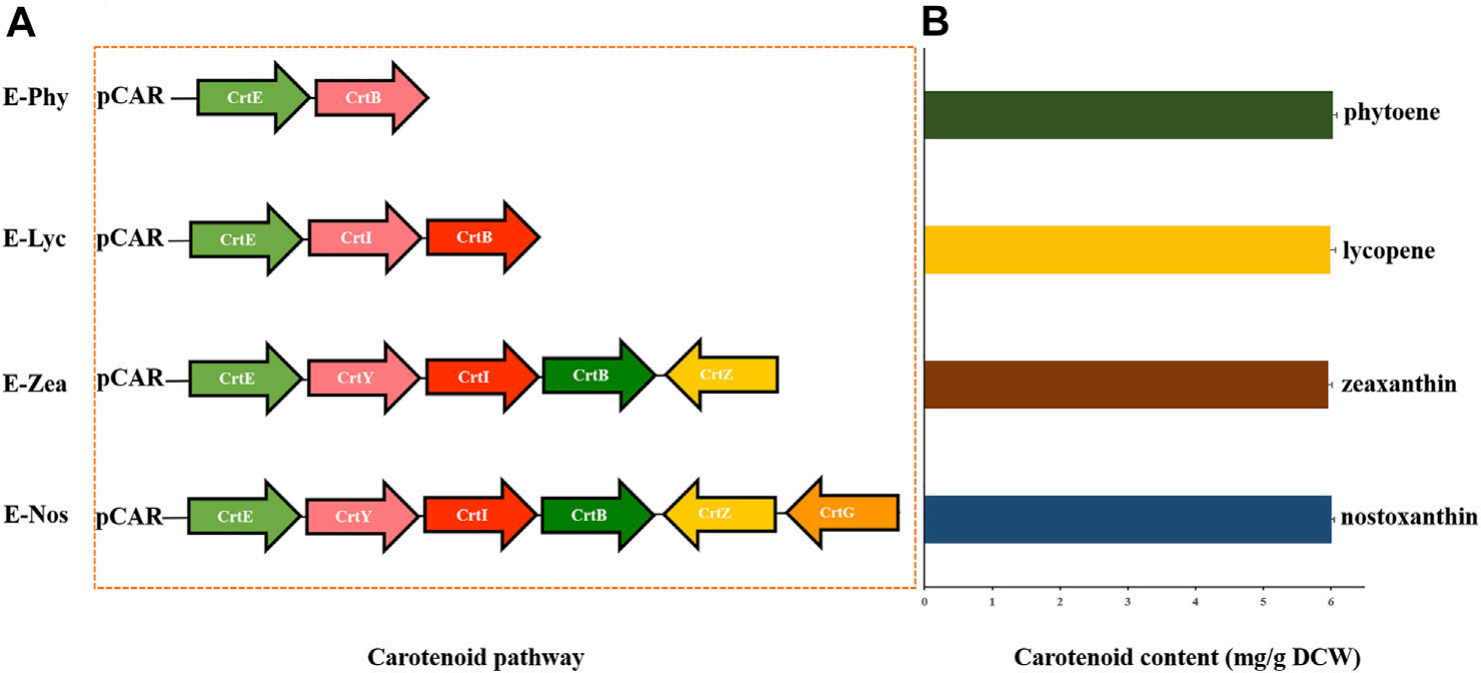
Engineered *Escherichia coli* JM109 expressing the genes for the production of specific carotenoids. **(A)** Plasmids containing the carotenogenic genes from *Sphingobium* sp. KIB (*CrtE*, *CrtB*, *CrtI*, *CrtY*, *CrtZ*, and *CrtG*). **(B)** Contents of carotenoids in engineered strains of E-Phy, E-Lyc, E-Zea, and E-Nos detected by ultra-performance liquid chromatography (UPLC).

For further confirmation of the catalytic efficiency of carotenoid biosynthetic genes in *Sphingobium* sp. KIB, a high-efficiency targeted metabolic engineering carotenoid biosynthesis platform was constructed in *E. coli* (Figure 5). According to bioinformatic analysis (Liu et al., 2019) and mutant analysis, six enzymes, namely, CrtE (geranylgeranyl pyrophosphate synthase), CrtB (phytoene synthase), CrtI (phytoene desaturase), CrtY (lycopene beta-cyclase), CrtZ (beta-carotene hydroxylase), and CrtG (2,2′-beta-ionone hydroxylase) (GenBank: no. SRR8864026), involved in the successive condensation process from IPP and DMAPP to nostoxanthin, were identified. These engineered *E. coli* strains, named as E-phy, E-lyc, E-zea, and E-nos, were able to accumulate 6.01 ± 0.034 mg of phytoene, 5.96 ± 0.058 mg of lycopene, 5.99 ± 0.083 mg of zeaxanthin, and 6.03 ± 0.057 mg of nostoxanthin g^−1^ dry cell, respectively, when cultured in LB medium for 48 h (Figure 5B). From these analyses, we clarified the entire carotenoid biosynthesis pathway and found that the genes involved in carotenoid biosynthesis of *Sphingobium* sp. KIB, thus, provided more biosynthesis components for bioengineering of carotenoids.

### Metabolic Engineering of *Sphingobium* to Produce High-Titer Astaxanthin

**FIGURE 6:**
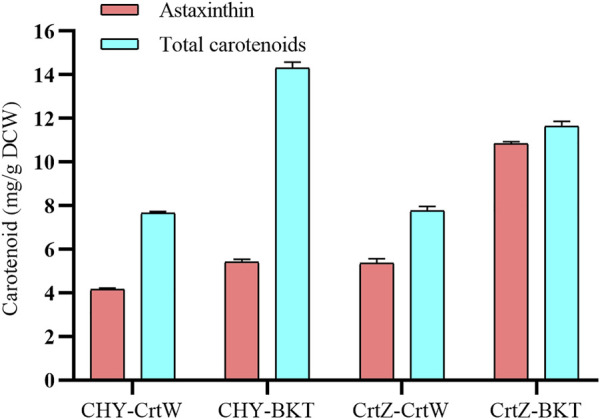
Engineered *Sphingobium* sp. KIB-zea combination expressing two β-carotene ketolase and two β-carotene hydroxylase for the production of astaxanthin. BKT from *Chlamydomonas reinhardtii*; CrtW from *Brevundimonas* sp. SD212; CHY from *Haematococcus pluvialis*; CrtZ from *Brevundimonas* sp. SD212.

Some noncarotenogenic bacteria and yeast have been engineered to accumulate astaxanthin by heterologous expression of a series of carotenogenic genes. However, the yield is less than unsatisfactory compared with other carotenoids (Jin et al., 2018). To further prolong the metabolic pathway, we engineered *Sphingobium* sp. KIB-zea to accumulate astaxanthin. A previous study suggested that the pathway from β-carotene to astaxanthin is the limited step of astaxanthin synthesis, which was catalyzed by β-carotene ketolase and β-carotene hydroxylase (Scaife et al., 2009). The combination of these two enzymes is critical to astaxanthin production. We combination express two β-carotene ketolase (*Chlamydomonas reinhardtii* BKT and *Brevundimonas* sp. SD212 CrtW) and two β-carotene hydroxylase (*Haematococcus pluvialis* CHY and *Brevundimonas* sp. SD212 CrtZ) to find a better combination for efficiently converting to astaxanthin in *Sphingobium* sp. KIB-zea. Among these four combinations, CrtZ and BKT was the best one with the highest titer of astaxanthin (10.75 mg/g DCW) and total carotenoids (11.6 mg/g DCW) (Figure 6). It should be pointed out that the high astaxanthin content in this strain represents 93% of total carotenoids, which will facilitate downstream processing to obtain a pure product.

Carotenoids are bioactive compounds with numerous biological functions. The increasing demand of various carotenoids has led to many methods to manufacture carotenoids. To date, commercial carotenoids are mainly produced through chemical synthesis. On an industrial scale, carotenoids can also be extracted from naturally producing organisms, e.g., astaxanthin from *Haematococcus pluvialis* (Lorenz and Cysewski, 2000), β-carotene from *Dunaliella* (Ben-Amotz, 1995), and *Blakeslea trispora* (Goksungur et al., 2002). In addition, heterologous biosynthesis of carotenoids in noncarotenogenic microorganisms, such as *E. coli* and *Saccharomyces cerevisiae*, has achieved great progress (Chen et al., 2013; Zhou et al., 2015; Shen et al., 2016). Bacterial cultivation is more convenient for large-scale production due to their unicellular nature and high growth rate (Silva et al., 2004; Shiloach and Fass, 2005). Hence, the noncarotenogenic bacterium *E. coli* has been engineered to produce significant amounts of carotenoids with different strategies. At least five heterologous genes were required to make *E. coli* produce zeaxanthin at a level of 1.1 mg g^−1^ dry cell weight (Misawa et al., 1990). Strategies, including coordinating the expression of two or more enzymes (Li et al., 2015), codon optimization (Wang et al., 2011), gene copy number adjustment (Misawa, 2011; Zelcbuch et al., 2013), and integrated multifactor (Li and Huang, 2018) were used to improve carotenoid production. Generally, two or more plasmids consisting of a number of genes had to be introduced into the host (Zelcbuch et al., 2013; Ma et al., 2016), or alternatively, the expression cassettes were integrated into the genomes of the targeted organisms (Zelcbuch et al., 2013; Lu et al., 2017). However, the introduction of too many foreign genes generally resulted in impediment of host growth and production instability (Shen et al., 2016). As a result, no engineered bacteria have been used for carotenoid production on a large scale.

Thus, carotenogenic microorganisms, in particular, those that are generally regarded as safe, e.g., sphingomonads species (Liu et al., 2012b; Kim et al., 2014; Ma et al., 2016), are promising hosts for carotenoid production. Our strain *Sphingobium* sp. KIB produced nostoxanthin up to 93% of the total carotenoids, which was much higher than that from others (Silva et al., 2004; Asker et al., 2009; Zhu et al., 2012), and demonstrated that *Sphingobium* sp. KIB could effectively use agro-industrial byproducts, such as glycerol, okara, and corn steep liquor, to benefit cell growth and stimulate carotenoid accumulation. Furthermore, we demonstrated that this strain could be tailored to specifically produce various carotenoids at a level of over 5 mg g^−1^ dry cell weight through chemical mutagenesis and produce astaxanthin at a level of 10 mg g^−1^ dry cell weight through directed metabolic engineering. So far, *Sphingobium* species have not been reported to be animal pathogens. Moreover, *Sphingobium* was found to possibly play a role in preventing acute otitis media (AOM) (Chonmaitree et al., 2017). Hence, *Sphingobium* sp. KIB and its mutants could further serve as novel hosts for improved carotenoid productivity by metabolic engineering of the endogenous carotenoid pathways reported by us recently (Liu et al., 2019).

## Conclusion

In conclusion, a new *Sphingobium* sp. was isolated and found that it could use agro-industrial byproducts, such as soybean meal, okara, and corn steep liquor, to accumulate large amounts of carotenoids. Then random mutagenesis or directed metabolic engineering was used to make the *Sphingobium* strain accumulate various high-value carotenoids, including phytoene, lycopene, zeaxanthin, and astaxanthin. Our study provided more biosynthesis components for bioengineering of carotenoids and highlights the potential of the industrially important bacterium for the production of various natural carotenoids.

## Data Availability

The original contributions presented in the study are included in the article/Supplementary Material, further inquiries can be directed to the corresponding authors.
