# Supplementary material for: Engineering Sphingobium sp. to Accumulate Various Carotenoids Using Agro-Industrial Byproducts
Source: Front Bioeng Biotechnol. 2021 Nov 4;9:784559. doi: 10.3389/fbioe.2021.784559 (PMC8600064; doi:10.3389/fbioe.2021.784559)
Supplement: Supplementary file 1 [file DataSheet1.docx]

Table S1 Primers used in this study.

| **Primer** | **Sequence** | **Products** | **PCR condition** |
| --- | --- | --- | --- |
| SPcrtEF | 5'-ATGGGGGGGGATGTTTCGTCG-3' | *crtE* | 2min 98℃; 10s 98℃; 5s 58℃; 30s 72℃; |
| SPcrtER | 5'-CGGCGATTTGGCGATATTGGTC-3' | *crtE* |  |
| SPcrtBF | 5'-ATGACCGACCTGCCCATATTCGAC-3' | *crtB* | 2min 98℃; 10s 98℃; 5s 61℃; 30s 72℃; |
| SPcrtBR | 5'-GGCCGTGGCACAGTTCAGACC-3' | *crtB* |  |
| SPcrtIF | 5'-ATGACCAGGAAAGCGATAATCATCG-3' | *crtI* | 2min 98℃; 10s 98℃; 5s 57℃; 30s 72℃; |
| SPcrtIR | 5'-CTAGCCCAGATCTTCCAGCATC-3' | *crtI* |  |
| SPcrtYF | 5'-ATGACAAACCGGTTGGACTGCGAT-3' | *crtY* | 2min 98℃; 10s 98℃; 5s 59℃; 30s 72℃; |
| SPcrtYR | 5'-TCAGGCGGGCGGATTCAGC-3' | *crtY* |  |
| SPcrtZF | 5'-ATGAACCTCGTCCTGCTCCTGCTG-3' | *crtZ* | 2min 98℃; 10s 98℃; 5s 59℃; 30s 72℃; |
| SPcrtZR | 5'-CGAGAAGGCCAAGACGATGAAGGT-3' | *crtZ* |  |
| SPcrtGF | 5'-ATGGTCGCAGCTATTCTTTTGTCCG-3' | *crtG* | 2min 98℃; 10s 98℃; 5s 57℃; 30s 72℃; |
| SPcrtGR | 5'-GGCATCCGGGTGACGCAGAC-3' | *crtG* |  |
| *CrtER* | 5‘-TTAACTGACGGCAGCGAGTT-3’ | pACCARcrtEB | 2min 98℃; 10s 98℃; 5s 56℃; 2min 72℃; |
| *CrtBF* | 5‘-ATAATCCGTCGTTACTCAATCA-3’ | pACCARcrtEB |  |
| *CrtER* | 5‘-TTAACTGACGGCAGCGAGTT-3’ | pACCARcrtEBI | 2min 98℃; 10s 98℃; 5s 56℃; 2min 72℃; |
| *CrtIF* | 5‘-TAAAGAGCGACTACATGAAACC-3’ | pACCARcrtEBI |  |


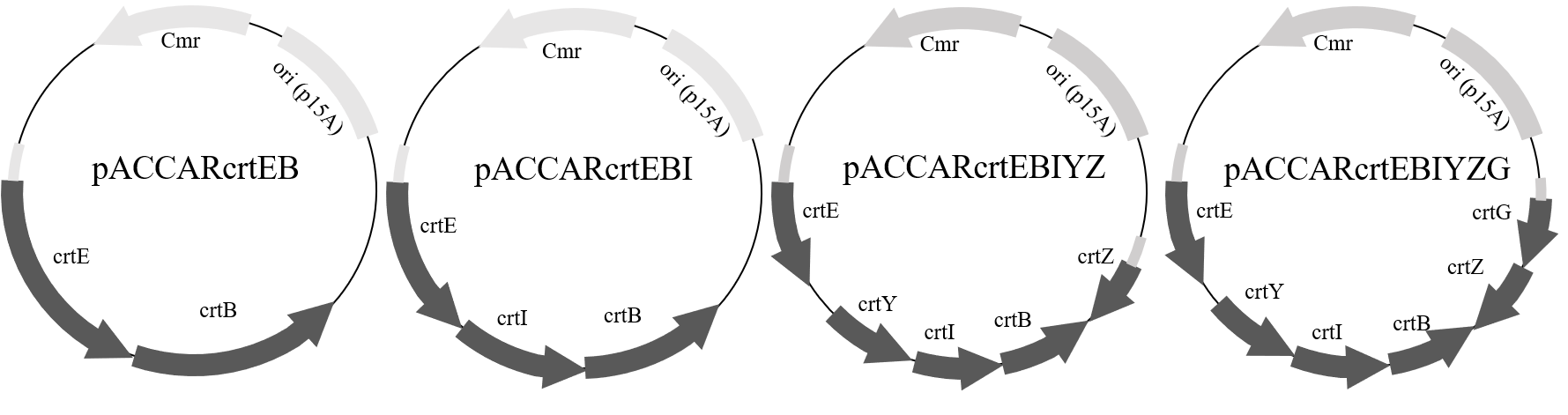


**Figure S1** The structures of plasmids. The replication origin is p15A. Plasmids pACCARcrtEB, pACCARcrtEBI, pACCARcrtEBIYZ, and pACCARcrtEBIYZG are derivatives of pACCAR16ΔcrtX.
